# pH-Mediated Single Molecule Conductance of Cucurbit[7]uril

**DOI:** 10.3389/fchem.2020.00736

**Published:** 2020-08-25

**Authors:** Qiushuang Ai, Qiang Fu, Feng Liang

**Affiliations:** ^1^The State Key Laboratory for Refractories and Metallurgy, School of Chemistry and Chemical Engineering, Wuhan University of Science and Technology, Wuhan, China; ^2^Jiangxi College of Traditional Chinese Medicine, Fuzhou, China

**Keywords:** recognition tunneling, single molecule conductance, pH-response, cucurbit[7]uril, melphalan, host-guest interactions

## Abstract

Recognition tunneling technique owns the capability for investigating and characterizing molecules at single molecule level. Here, we investigated the conductance value of cucurbit[7]uril (CB[7]) and melphalan@CB[7] (Mel@CB[7]) complex molecular junctions by using recognition tunneling technique. The conductances of CB[7] and Mel@CB[7] with different pH values were studied in aqueous media as well as organic solvent. Both pH value and guest molecule have an impact on the conductance of CB[7] molecular junction. The conductances of CB[7] and Mel@CB[7] both showed slightly difference on the conductance under different measurement systems. This work extends the molecular conductance measurement to aqueous media and provides new insights of pH-responsive host-guest system for single molecule detection through electrical measurements.

## Introduction

Molecular electronics, which focuses on the single molecules in the electronic junctions used to make electronic devices, has been extensively studied. Molecular electronics aims at investigating single molecules at electrical junctions for fabricating electronic devices, which has been extensively investigated (Cui, 2001; Choi and Mody, 2009; Herrer et al., 2018). A variety of methods have been developed to measure the electrical properties of single molecules by constructing metal-molecule-metal junctions, including scanning probes techniques such as scanning tunneling microscopy (STM) techniques (Xu and Tao, 2003; Haiss et al., 2006; Venkataraman et al., 2006; Chen et al., 2020; Yu et al., 2020), conducting atomic force microscopy (AFM) (Cui, 2001), mechanically controlled break junctions (MCBJ) (Reed et al., 1997; Smit et al., 2002), and nanoparticle dimers (Dadosh et al., 2005; Fernandez et al., 2014).

Considering possible contaminations in the air and electrochemical leakage current in aqueous media, STM measurements are normally performed in organic solvent system. This limited the applications in biomedical field to a certain extent. The local molecular environment should be taken into account when measuring single-molecule electrical properties in the design of single molecule sensing devices (Zhang et al., 2016).

Molecular interactions included some dynamic behaviors in biological systems can be greatly influenced by various stimuli, such as pH, temperature, light, and so on (Angelos et al., 2008; Mendes and Paula, 2008; Yu et al., 2015). Stimuli-responsive host-guest interactions has been established in solution or on surfaces, and normally studied by traditional analytical methods like nuclear magnetic resonance (NMR), fluorescence, and isothermal titration calorimetry (ITC), etc. (Cabane et al., 2012; Yang et al., 2014a,b; Sinn and Biedermann, 2018; Xiao et al., 2019). However, electrical properties of pH-responsive supramolecular systems are rarely investigated by recognition tunneling technique.

Belong to a family of macrocyclic host molecule, barrel-shaped cucurbit[n]uril (CB[n]s) has gained much interest for applications including sensors, nanoreactors and drug delivery due to its good biocompatibility, high thermal stability and remarkable recognition properties (Macartney, 2011; Ma and Zhao, 2015; Kuok et al., 2017; Gao et al., 2018; Yin and Wang, 2018; Zhang et al., 2018; Braegelman and Webber, 2019; Cheng et al., 2020). CB molecule can be immobilized on the gold surface through the collective interactions between multiple carbonyl groups of CB and gold (An et al., 2008). CB[n] retains its recognition properties when bonded to the gold surface, making it a promising candidate for molecular sensors. In addition, the introduction of CB into the conductance measurement system provides a platform to investigate the molecules that cannot tether with gold. Here, we chose CB[7] as the host molecule to explore the electrical properties via STM fixed junction technique. CB[7] is known with good water solubility and high binding affinity with various guest molecules, and only one guest molecule can be confined inside its cavity at a given time.

CB[7] can allow the inclusion of melphalan (Mel) in its cavity. As an antineoplasic drug, Mel is indicated for the treatment of multiple myeloma and other types of cancer (Samuels and Bitran, 1995; Falco et al., 2007). While the usability of Mel is limited to its poor water solubility at neutral pH as well as rapid hydrolysis in physiological conditions. However, the solubility and stability of Mel can be efficiently improved upon introducing CB molecule, which promotes further application for therapy (Zhang and Isaacs, 2014; Villarroel-Lecourt et al., 2018). Notably, the binding affinity between Mel and CB[7] differs as the pH changes (Villarroel-Lecourt et al., 2018). This makes Mel@CB[7] complex a potential pH-responsive sensor.

We investigated the conductances of CB[7] and its host-guest complex (Mel@CB[7]) with different pH values by using STM fixed junction technique. CB[7] and Mel@CB[7] functionalized gold substrates were prepared by immersing gold substrates into various pH phosphate buffer solution (PB) containing CB[7] and Mel@CB[7], respectively. Both CB[7] and Mel@CB[7] showed differences on the conductance when pH value changed. In addition, we compared the conductance measurements performed in PB and organic solvent 1, 2, 4-trichlorobenzene (TCB). Slight difference on the conductance was observed under different measurement systems. This work extends the single molecule conductance measurements from normal organic system to aqueous media for investigating dynamic molecular interactions and aims at exploring potential application for fabricating pH-responsive supramolecular system for single molecule detection as well as the potential application for drug delivery.

## Results and Discussion

**Figure 1:**
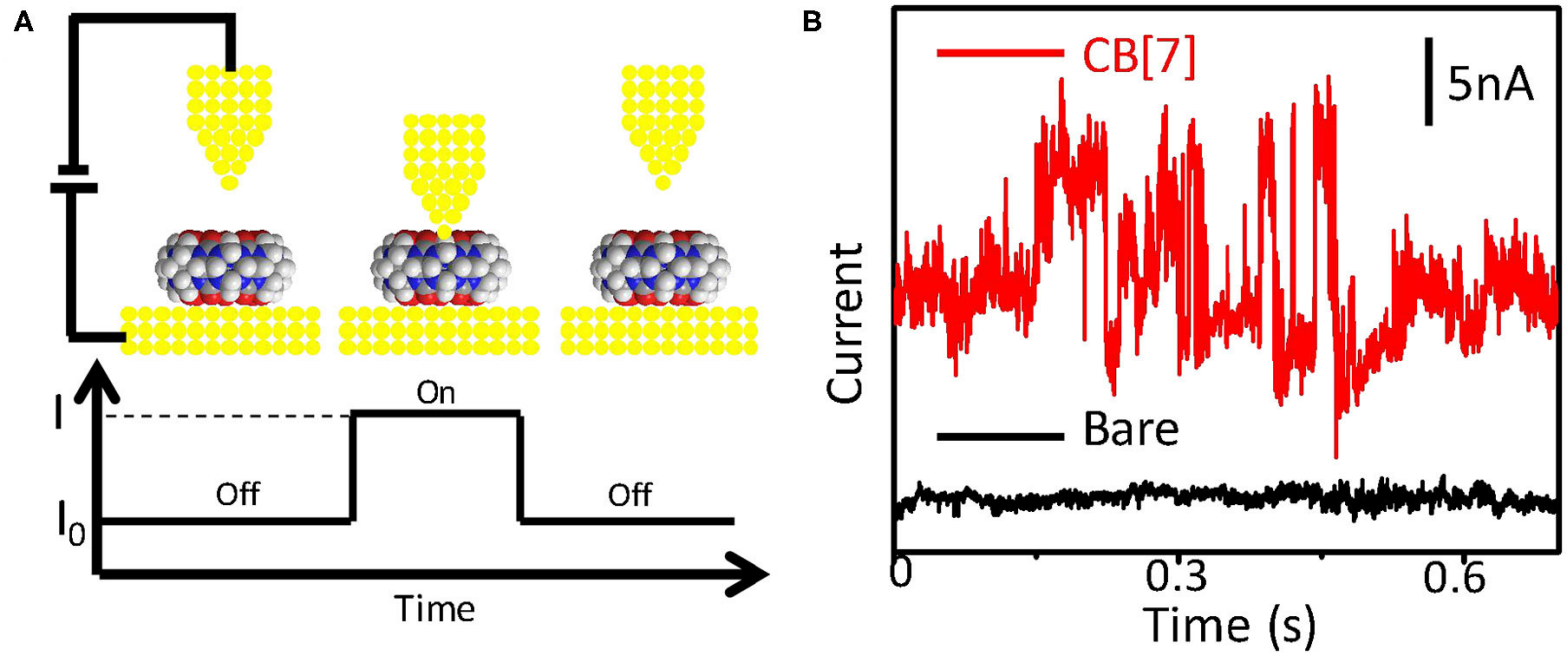
Experimental setup for conductance measurement. **(A)** Schematic diagram of the conductance measurement of CB[7] (top), and the spontaneous formation of a molecular bridge between the tip and the substrate (bottom). **(B)** Typical current traces for the substrate before (black) and after (red) modified with CB[7] for overnight. Measurements were carried out with a sample bias of 0.1 V and a set-point current of 20 nA.

The schematic setup of the conductance measurement of CB[7] is shown in Figure 1A. The conductance measurement method is based on the STM fixed-junction technique. Generally, a gold probe is placed in close proximity to gold surface functionalized with the target molecule where the distance between the probe and the substrate is fixed at a few tens of nanometers controlled by the piezoelectric transducer (PZT) of a STM. The target molecules are not disturbed by electrodes moving or crashing into each other, which makes it suitable for the study of noncovalent interactions between single molecules (Chen et al., 2020). No signal was observed on clean gold substrate (black curve of Figure 1B) or on gold surface modified with CB[7] where no effective molecular junctions form (“off” state). The target molecule on the substrate contacts the probe to form a molecular bridge, giving rise to the current jumps (“on” state) (Haiss et al., 2004, 2006). The current fluctuations represent the fast formation and breaking of molecular bridge. With the carbonyl group of CB[7] molecule on the surface binding to both electrodes, current spikes occurred (red curve of Figure 1B). These current spikes indicated the transient formation of Au-CB[7]-Au molecular junction. The time-dependent STM image of CB[7] functionalized gold substrate was shown in Figure S1A. The appearance of pits in the STM image suggested that CB[7] can interact with the gold substrate to relocate the surface gold atoms (Xiao et al., 2018), which indicated the successful modification of CB[7]. This was further proven by the decrease of contact angle after modifying CB[7] (Figure S1B).

## Single Molecule Conductance Measurements of CB[7]

We first investigated the single-molecule conductance and bonding lifetime of CB[7] molecular junction where measurements were carried out in PB solution. The gold probes used under this condition were insulated to rule out the electrochemical leakage current. Freshly cleaned gold substrates were immersed in PB buffer containing CB[7] molecules with various pH values for overnight prior to the conductance measurements. The pH values tried here were 1, 4, and 7.

The single molecule conductance of CB[7] can be determined by transient current changes (Haiss et al., 2004; Xiao et al., 2018).

$$\begin{array}{l} G=\frac{\left(I-I_{0}\right)}{V} \end{array}$$

**Figure 2:**
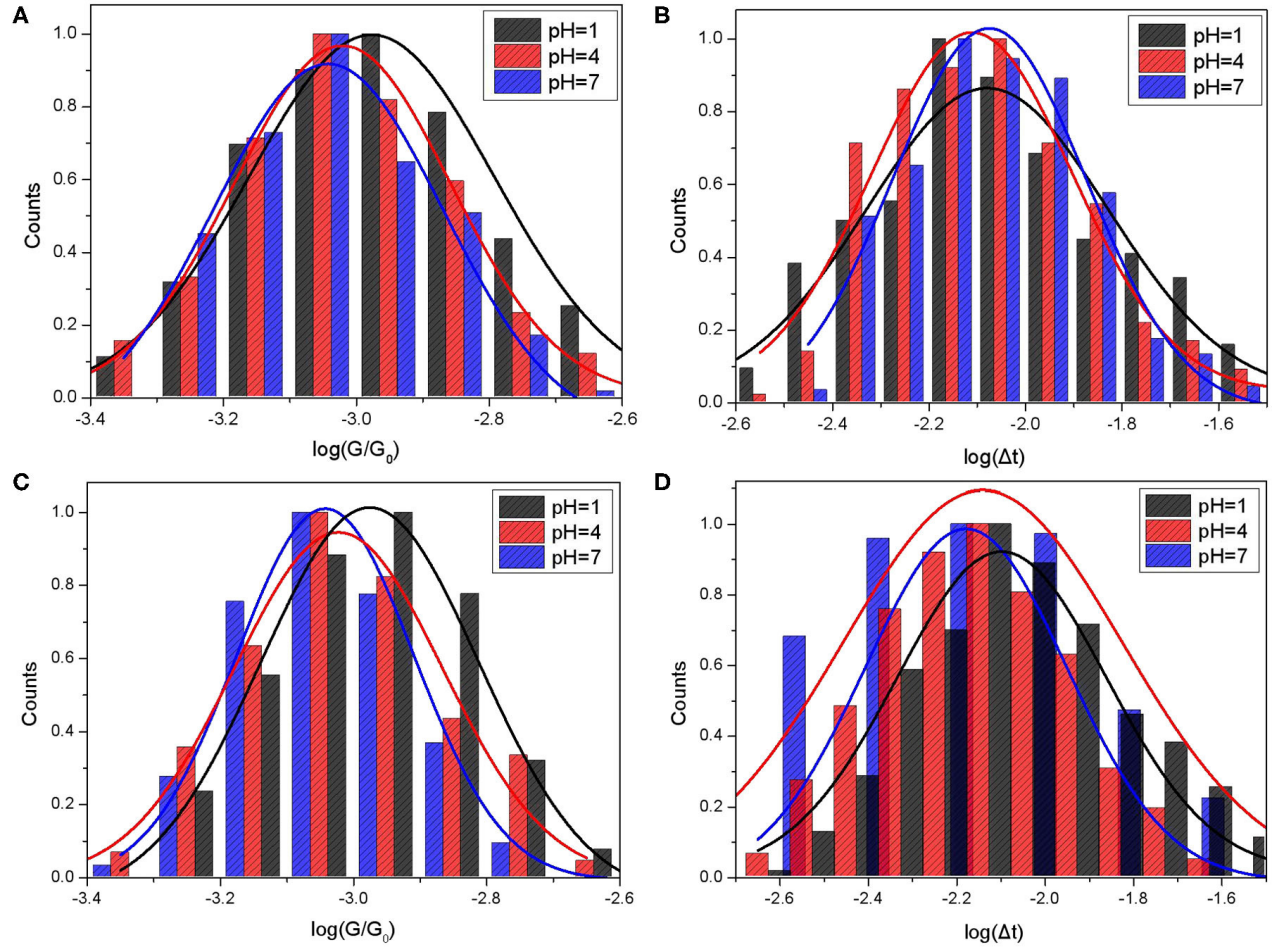
Single molecule conductance and bonding lifetime of CB[7] molecular junction. The molecule conductance histograms **(A,C)** and lifetime histograms **(B,D)** of switching events for CB[7] were plotted in logarithm scale. Gold substrates were modified with CB[7] in PB buffer solution with the pH values at 1, 4, and 7. STM measurements were performed in PB **(A,B)** and TCB **(C,D)** at a constant bias of 0.1 V with the setpoint of 20 nA.

Where *I* is current, *I*_0_ is the tunneling current before the observation of the current jump, V is the applied bias. In this report, the baseline current *I*_0_ is 20 nA, and the bias V is 0.1 V, which follows the previous report (Xiao et al., 2018). The histograms of conductance G vs. G_0_ were plotted in semi-log scale, where G_0_ is quantum of conductance 77.4 μS, as shown in Figure 2A (in PB) and C (in TCB). The corresponding results are displayed in Table 1.

**Table 1:** Single molecule conductance and lifetime of switching events for CB[7].

	**pH**	**G (nS)**	**Lifetime (ms)**
PB	1	81.61 ± 20.03	8.33 ± 3.86
PB	4	73.75 ± 25.04	7.74 ± 3.09
PB	7	71.26 ± 30.31	8.41 ± 2.95
TCB	1	73.58 ± 33.16	7.52 ± 3.63
TCB	4	71.08 ± 30.52	7.68 ± 3.70
TCB	7	70.27 ± 37.64	7.23 ± 4.34

Based on the Gaussian fit, the mean conductance of CB[7] measured in PB were 81.61 nS, 73.75 nS, and 70.26 nS for pH 1, 4, and 7, respectively. The conductance gradually decreased as the pH value increased. More protons would interact with carbonyl group under acid condition, which may contribute to the electron transfer of CB[7] molecular junction. Meanwhile, we also checked the width of the switching spike, giving the information of bonding stability between CB[7] molecule and gold electrodes. The histogram and Gaussian fit of the switching lifetime Δt, plotted in log scale was shown in Figure 2B. The mean value of switching events for CB[7] was between 7 and 8 ms, which indicated the short-time stability of the binding geometry associated with the collective interaction between single or few carbonyl groups of CB[7] and gold electrode.

We also studied single-molecule conductance measurements of CB[7] performed in TCB. The histograms and Gaussian fit of the switching events for CB[7] measured in TCB are shown in Figures 2C,D. Based on the results in Table 1, the mean conductance of CB[7] measured in TCB were 73.58 nS, 71.08 nS, and 70.27 nS for pH 1, 4, and 7, respectively. Just like the conductance measured in PB, the conductance here showed slightly decrease as the pH value increased. To confirm the pH effect, we further investigated the conductance of CB[7] where the pH was 9 (Figure S2). The mean conductance of CB[7] measured in PB and TCB were 64.52 nS and 63.2 nS, respectively (Table S1). The conductance of CB[7] at pH 9 was smaller than that at pH 7, which further indicated the interactions between protons and carbonyl group affected the electron transfer process of the CB[7] molecular junction.

In contrast to the lifetime and the conductance of CB[7] measured in PB, the values measured in TCB were smaller under the same pH value, especially for pH 1. This suggested the interactions between protons and carbonyl group under aqueous condition may promote better electron transfer to a certain extent.

## Single Molecule Conductance Measurements of Mel@CB[7]

**Figure 3:**
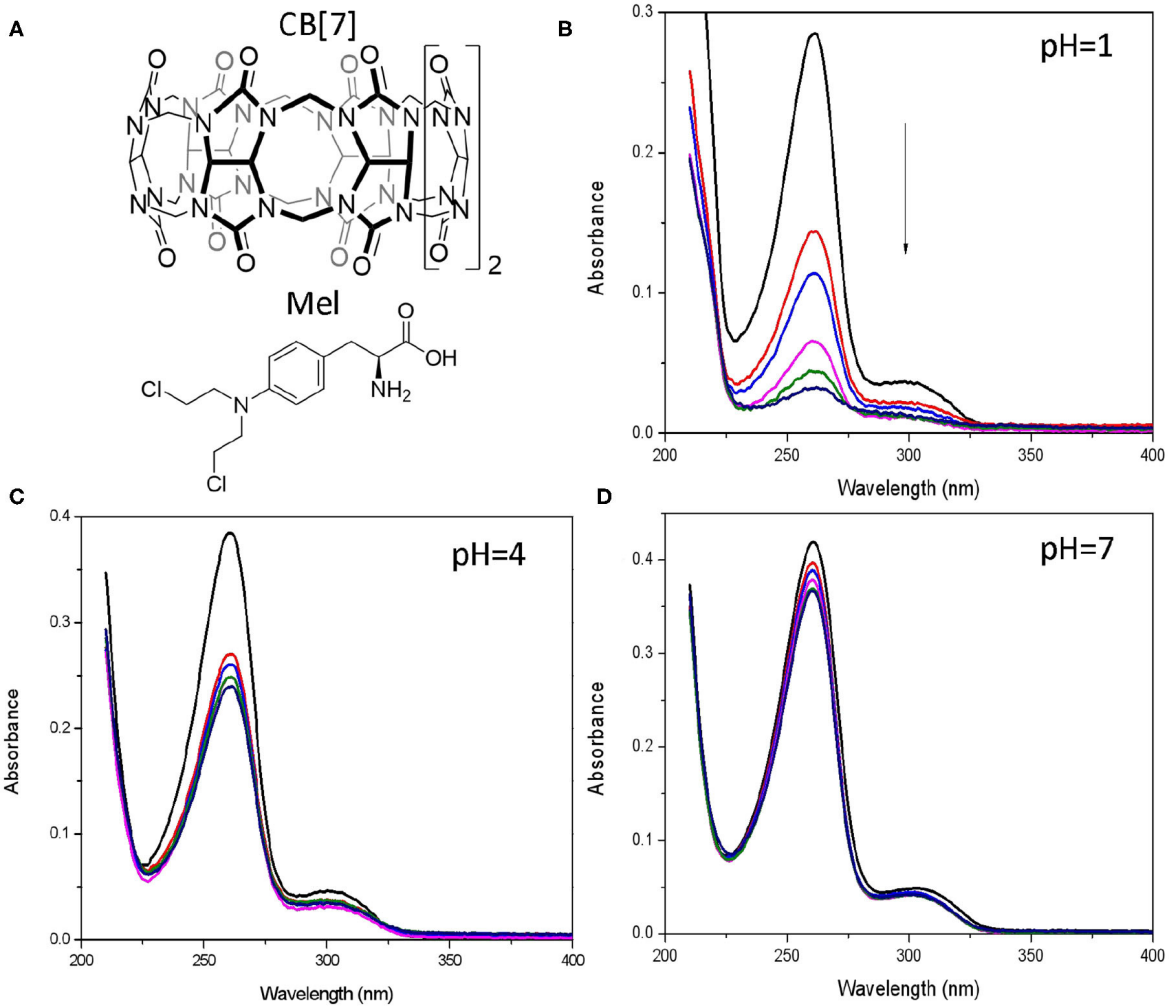
Spectroscopic characterization for Mel@CB[7] interaction. **(A)** Chemical structure for CB[7] and Mel. **(B–D)** UV-Vis absorption of Mel (20 μM) upon addition of CB[7] (0–40 μM) in 10 mM phosphate buffer (**B**, pH = 1, **C**, pH = 4, **D**, pH = 7).

We further explored the conductance of CB[7] with melphalan encapsulated inside its cavity. The chemical structure of CB[7] and Mel are shown in Figure 3A. Mel has been reported to form 1:1 host-guest complex with CB[7], with the equilibrium binding affinity log K = 6 at pH 1 (Villarroel-Lecourt et al., 2018). The protonation of Mel is essential for binding to the macrocycle due to the cation-dipole interactions. We first studied the pH value effect on the interactions between Mel and CB[7] through absorption spectroscopy.

UV/vis spectra of Mel showed a remarkable decrease in the absorption band at 260 nm at pH 1 in the presence of increasing concentrations of CB[7] (Figure 3B), which attributed to the encapsulation of the drug. Slightly decrease for the absorption spectra of Mel was observed at pH 4 with the increasing of CB[7] (Figure 3C). Interestingly, this change was not observed at pH 7 (Figure 3D) because of the weak host-guest interaction of the complex at this pH, which is also consistent with the simulation of Mel@CB[7] complex in the docking study (Villarroel-Lecourt et al., 2018). Considering the rapid hydrolysis of Mel as well as the lack of obvious binding between Mel and CB[7] under alkaline conditions, subsequent conductance measurements focused on pH values at 1, 4, and 7.

Similar to CB[7]'s immobilization, Mel@CB[7] complexed molecules were deposited onto the gold substrate prior to conductance measurements. CB[7] here served as a nanocontainer to immobilize the guest molecule for STM measurements. No single molecule conductance event was observed for Mel due to no effective molecular junction forming.

**Figure 4:**
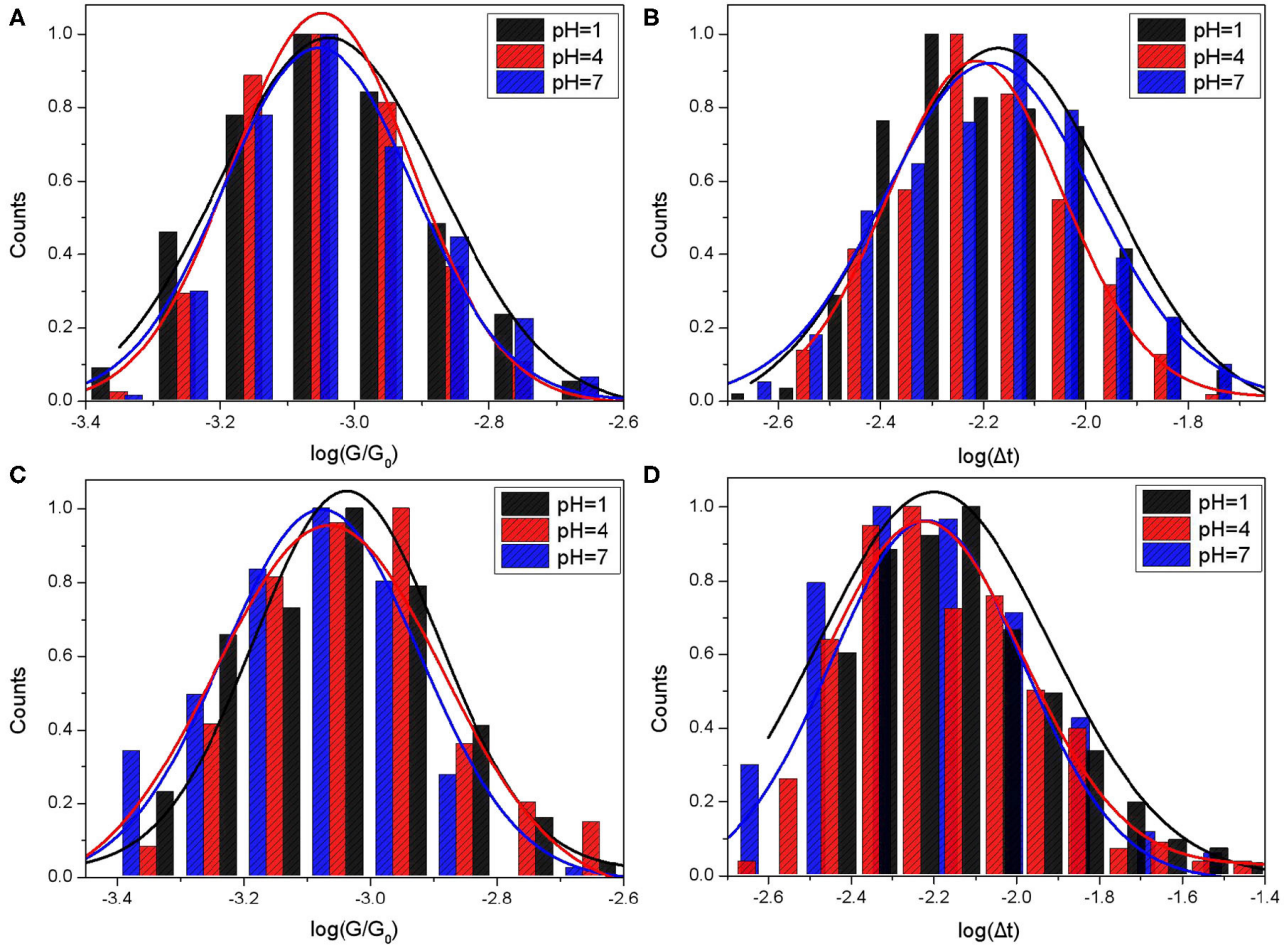
Single molecule conductance and bonding lifetime of Mel@CB[7] molecular junction. The molecule conductance histograms **(A,C)** and lifetime histograms **(B,D)** of switching events for Mel@CB[7]. Gold substrates were modified with Mel@CB[7] PB buffer solution where the pH values were 1, 4, and 7. STM measurements were performed in PB buffer **(A,B)** and TCB **(C,D)** at a bias of 0.1 V with the setpoint 20 nA.

The histograms and Gaussian fit of G vs. G_0_ and the lifetime Δt in semi-log scale for Mel@CB[7] complex are shown in Figure 4, where measurements were performed in PB (A, B) buffer and TCB (C, D), respectively. The responding results are summarized in Table 2.

**Table 2:** Single molecule conductance and lifetime of switching events for Mel@CB[7].

	**pH**	**G (nS)**	**Lifetime (ms)**
PB	1	71.41 ± 31.89	6.74 ± 3.24
PB	4	69.30 ± 37.38	6.11 ± 2.50
PB	7	68.35 ± 36.11	6.50 ± 2.97
TCB	1	69.08 ± 35.05	6.09 ± 3.17
TCB	4	66.64 ± 27.78	6.08 ± 3.64
TCB	7	64.82 ± 33.39	6.41 ± 4.41

As shown in Table 2, the mean conductance of Mel@CB[7] complex measured in PB were 71.41 nS, 69.3 nS, and 68.35 nS for pH 1, 4, and 7, respectively. The conductances of Mel@CB[7] measured in PB gradually decreased with the increase of pH value, which is similar to the change trend of CB[7]. However, it is worth noting that compared with CB[7], the Mel@CB[7] complex showed a smaller conductance at the same pH. Guest molecule in the cavity of CB[7] had an impact on electronic transport process, which is consistent with the previous report (Xiao et al., 2018). Furthermore, the mean lifetime of the switching events for Mel@CB[7] was between 6 and 7 ms, which was smaller than that for CB[7]. This suggested that the inclusion of Mel inside CB[7]'s cavity weakened the bonding stability between CB[7] molecule and gold electrodes.

The mean conductance of Mel@CB[7] measured in TCB were 69.08 nS, 66.64 nS, and 64.82 nS for pH 1, 4, and 7, respectively. The conductance here also slightly decreased as the pH value increased. As for the measurements performed in TCB, both the mean conductance and the mean lifetime of Mel@CB[7] complex were smaller than that of CB[7] under the same pH value. This further indicated the guest molecule in the cavity of CB[7] disturbed the electron transport process, as well as the bonding stability between CB[7] molecule and gold electrodes.

However, compared with the conductance measured in PB, only a slight decrease of the conductance at pH 1 was observed for Mel@CB[7] complex when the measurement was performed in TCB. This may further indicate that the guest molecule in the cavity play a dominant role in disturbing the electron transfer in the molecular junction.

## Materials and Methods

### Chemicals

Cucurbit[7]uril (CB[7]) was synthesized following a procedure published by Day's group (Day et al., 2001). Gold wire, sodium chloride, sodium dihydrogen phosphate, dibasic sodium phosphate, sodium hydroxide, hydrochloric acid and absolute ethanol were purchased from Sinopharm Chemical Reagent Co., Ltd. Melphalan was purchased from aladdin. 1, 2, 4-trichlorobenzene was purchased from Sigma-Aldrich. All the purchased chemicals were used directly without further purification. Deionized water from a Milli-Q water purifying system was used to prepare all of the solutions (18.2 MΩ).

### STM Probes Fabrication

STM probes were fabricated by following previous report (Tuchband et al., 2012). A gold wire was electrochemically etched and cleaned by dipping into piranha solution (H_2_SO_4_:H_2_O_2_ = 3:1, by volume), rinsed with copious amounts of deionized water, and dried with argon. This gold tip was directly used in the STM measurements carried out in organic solvent. For the measurements performed in PB buffer, the gold tip was further insulated with high-density polyethylene (HDPE) to expose only the tip apex.

### Gold Substrates Preparation

10 mM phosphate buffer solution with pH 7 was prepared by mixing equal concentration of dibasic sodium phosphate solution and sodium dihydrogen phosphate solution (10 mM). PB buffer with pH 1, 4 and 9 were prepared by dropping hydrochloric acid and sodium hydroxide solution into pH 7 PB buffer, respectively. A 1 mM CB[7] solution was prepared by dissolving CB[7] powders in PB buffer with sonication for 5 min. A 1 mM Mel@CB[7] complex solution was prepared by mixing Mel powders in the CB[7] solution, followed by ~15 min sonication. Freshly cleaned gold substrates were immersed in molecular solution for overnight at room temperature. The modified gold substrates were washed with copious amounts of ethanol and deionized water, dried with argon gas, and used immediately.

### Recognition Tunneling Measurements

Recognition Tunneling measurements were performed on a STM (Keysight 5,500) with the sample and probe submerged in a liquid cell containing 1, 2, 4-trichlorobenzene or PB buffer at a bias of 0.1 V with the setpoint 20 nA. Current-time traces were recorded with a Picoview software. For each sample, 2~3 gold probes were used to collect hundreds of experimental runs over different sample positions. The PZT servo response time was about 30 ms, as described previously (Xiao et al., 2018). Current spikes were analyzed by home-built Labview programs.

### UV-Vis Measurements

10 mM PB buffer with pH 1, 4, 7 were prepared. A 20 μM Mel solution was prepared by dissolving Mel powders in PB buffer, following with sonication for 10 min. The association of Mel to CB[7] (0-40 μM) was measured by absorption spectroscopy (UV-3600, Japan).

### Contact Angle of SAMs

Water contact angles were measured using a goniometer (OCA15EC, Germany) immediately after the addition of 5.0 μL of water droplets on gold substrate.

## Conclusions

In summary, we investigated the statistical conductance value of CB[7] and its host-guest complex molecular junctions by using STM fixed junction technique. The conductances of CB[7] and Mel@CB[7] complex with different pH value were determined in PB buffer and organic solvent TCB. Both CB[7] and Mel@CB[7] complex showed decreased conductance as the pH value increased. This suggested that the pH affected the electron transfer process of the molecular junction. The mean conductance and the mean lifetime of Mel@CB[7] complex were smaller than that of CB[7] under the same pH value, which indicated that the encapsulation of Mel molecule inside CB[7]'s cavity disturbed the electron process and weakened the electronic coupling between host molecule and gold electrodes. In addition, the values of the lifetime and the conductance for both CB[7] and Mel@CB[7] measured in PB were greater than that in TCB under the same pH value, suggesting the interactions between protons and carbonyl group under aqueous condition promoting better electron transfer to a certain extent. We believed this work can contribute to exploring multi-functional stimuli-responsive supramolecular system for single molecule detection.

## Data Availability Statement

All datasets generated for this study are included in the article/Supplementary Material.

## Author Contributions

The project was conceptually designed by FL. The majority of the experiments and data analysis were carried out by QA. The manuscript was prepared and revised by QA, QF, and FL. All authors discussed the results and implications and commented on the manuscript.

## Conflict of Interest

The authors declare that the research was conducted in the absence of any commercial or financial relationships that could be construed as a potential conflict of interest.
