# Supplementary material for: pH-Mediated Single Molecule Conductance of Cucurbit[7]uril
Source: Front Chem. 2020 Aug 25;8:736. doi: 10.3389/fchem.2020.00736 (PMC7477741; doi:10.3389/fchem.2020.00736)
Supplement: Supplementary file 1 [file Data_Sheet_1.docx]

Supplementary Material

pH-Mediated Single Molecule Conductance of Cucurbit[7]uril

Qiushuang Ai^1^, Qiang Fu^2^, Feng Liang^1*^

^1^The State Key Laboratory for Refractories and Metallurgy, School of Chemistry and Chemical Engineering, University of Science and Technology, Wuhan, China

^2^Jiangxi College of Traditional Chinese Medicine, Fuzhou, China

*** Correspondence:** Feng Liang, feng_liang@wust.edu.cn

**Figure S1.** STM and contact angle characterization of CB[7] functionalized gold substrate. (A) STM image of the Au(111) substrate immersed in CB[7] solution for overnight. (B) Contact angle of water droplets on bare gold substrate (i) and after immersion with CB[7] (ii).

**Figure S2.** The molecule conductance histograms and lifetime histograms of switching events for CB[7] molecular junction. Gold substrate was modified with CB[7] PB buffer solution where the pH value was 9. STM measurements were performed in PB buffer (A, B) and TCB (C, D).

**Table S1.** **Single molecule conductance and lifetime of CB[7]**

|  | **pH** | **G (nS)** | **Lifetime (ms)** |
| --- | --- | --- | --- |
| PB | 9 | 64.52 ± 38.61 | 7.38 ± 2.89 |
| TCB | 9 | 63.20 ± 34.09 | 7.46 ± 3.63 |
